# Berries and Steps: a protocol of a randomized, placebo-controlled pilot study testing freeze-dried blueberry powder in sedentary older adults with mild depressive symptoms

**DOI:** 10.1186/s12937-025-01154-0

**Published:** 2025-05-29

**Authors:** Courtney L. Millar, Alex Wolfe, Kathryn Baldyga, Alyssa B. Dufour, Lewis A. Lipsitz

**Affiliations:** 1 Department of Medicine, Beth Israel Deaconess Medical Center, Harvard Medical School, Boston, MA USA; 2 Hinda and Arthur Marcus Institute for Aging Research, 1200 Centre St, Boston, MA 02131 USA

**Keywords:** Anthocyanins, Fiber, Aging, Inflammation, Depression, Microbiome, Flavonoids

## Abstract

**Background:**

Older adults spend the majority of their day engaging in sedentary behavior, which increases risk of mortality by 22%. Despite the well-established health benefits of physical activity, a large portion of older adults remain sedentary. Recent evidence suggests that inflammation contributes to lack of motivation, which is a critical barrier to overcoming sedentary behavior in older adults. Given that inflammation is highly modifiable by diet, an anti-inflammatory dietary strategy may be a viable way to improve inflammation-driven lack of motivation and ultimately increase physical activity in sedentary older adults. However, interventions targeting such a pathway are scarce. We propose a study intervention protocol, which aims to determine the feasibility and preliminary efficacy of daily supplementation of freeze-dried blueberries. Supplementation with blueberries provides 2 anti-inflammatory nutrients (fiber and anthocyanins) to theoretically reduce inflammation-driven lack of motivation and thereby enhance physical activity in older adults with sedentary behavior and mild depressive symptoms.

**Methods:**

The current study is planned as a single-site, randomized, double-blind, parallel pilot study in 40 older adults with sedentary behavior and mild depressive symptoms. Individuals with depressive symptoms often lack motivation and have increased levels of inflammatory cytokines, representing an ideal population for an anti-inflammatory dietary intervention to improve motivation. Participants will be randomized to consume either 48 g of freeze-dried blueberry powder (~ 600 mg of anthocyanins and ~ 8 g of fiber) or a nutritionally matched placebo powder (without any known amounts of anthocyanins and fiber) each day for a total of 12 weeks.

**Discussion:**

Identification of a dietary intervention to target the inflammatory pathways may offer a novel and feasible approach to increase motivation and engagement of physical activity in older adults. If feasible and effective, such a strategy would help avoid the plethora of health consequences associated with sedentary behavior and physical inactivity.

**Trial registration:**

The current study is approved by the Advarra IRB (#Pro00064749) and registered at Clinicaltrials.gov (Identifier: NCT05735587).

## Background

Sedentary behavior is associated with serious health consequences, including higher risk of mortality [1–8]. Thus, sedentary behavior is a pressing public health concern that has direct impact on the well-being of the aging population. For a healthy older adult, a step count between 7,000–10,000 steps per day is recommended, which theoretically indicates the individual is meeting the recommendations for moderate/vigorous exercise [9]. However, most individuals do not meet that recommendation. Although there are several determinants of sedentary behavior in older adults, lack of motivation to engage in physical activity is a significant contributor [10]. Thus, one strategy to reduce sedentary behavior is to first change the motivation to engage in physical activity.

Markers of inflammation have been consistently linked with lack of motivation [10–12]. In fact, administration of a low-dose endotoxin that increases inflammatory cytokines resulted in depressed mood and decreased brain activity in the ventral striatum during a monetary reward task compared to placebo [13]. Such a change in brain activity is indicative of anhedonia, or the lack of pleasure or motivation. This study suggests a direct link between inflammation, brain activity, and mood/behavior. It is hypothesized that inflammation may modulate brain activity and motivation through dopamine. Dopamine is a neurotransmitter in the brain that plays a critical role in behavior, particularly reward behavior and motivation [14]. Dopamine is especially important in the ventral striatum. Under usual circumstances, higher level of dopamine release during a behavior theoretically reinforces and individual’s motivation to re-engage in that behavior. However, in a state of inflammation, dopamine may not function appropriately. In depressed individuals, who typically experience a lack of motivation and have higher levels of inflammation, dopamine synthesis and release is impaired [15]. Furthermore, inflammatory cytokines circulating in the blood have been shown to modulate dopamine activity, which may result in lack of motivation [16]. Therefore, targeting inflammation holds great promise to increase motivation, potentially through dopaminergic pathways, in sedentary older adults and improve the downstream effects on engagement in physical activity.

It is well-known that inflammation is highly modifiable by diet [17–21]. Certain dietary constituents, such as anthocyanins and fiber, have been reported to have anti-inflammatory properties and lower inflammation. Anthocyanins are pigments found in fruits and vegetables that are responsible for the blue/purple colors [22]. Fiber is a non-digestible carbohydrate that helps sustain a healthy gut environment. Both anthocyanins and dietary fiber have anti-inflammatory properties, [21, 23–29] and nature has conveniently packaged both promising dietary constituents into one vessel: blueberries. Thus, blueberry consumption, as a daily source of anthocyanins and fiber, may be a viable strategy to lower inflammation, improve motivation, and reduce sedentary behavior in older adults.

The aim of this study is to gather preliminary evidence on the feasibility and preliminary efficacy of an intervention supplementing blueberries (as a source of fiber and anthocyanins) daily for 3 months compared to control (placebo) in older adults with sedentary behavior and mild depressive symptoms. Evaluated outcomes include recruitment rate, retention rate, compliance, markers of inflammation, and motivation, while the primary outcome of the study is physical activity engagement which we define as median daily step count.

## Materials and methods

### Study design

**Fig. 1:**
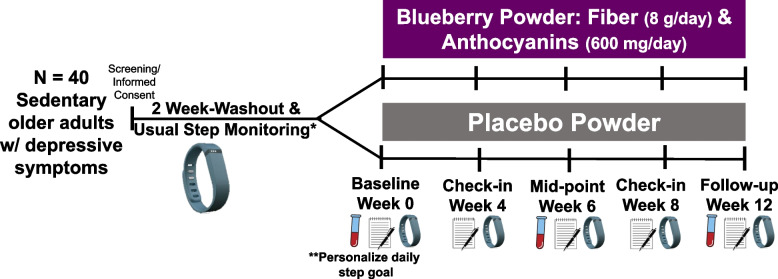
Pilot study design. This study aims to recruit 40 older adults with sedentary behavior and minor levels of depressive symptoms. Eligible participants will enter a 2-week washout period where they will avoid fiber- and anthocyanin-rich foods. During this time they will wear an accelerometer (i.e., ActiGraph GT9X Link) that measures their usual daily step count. At baseline participants will provide blood sample, fill out questionnaires, and continue to wear their ActiGraph for the next 12 weeks while also consuming their randomly assigned powder. **At the baseline visit, all participants will be provided a personalized daily step goal (defined as 20% increase from washout daily step median)

This will be an individual-level, double-blind, randomized, parallel-arm, pilot study in older adults with sedentary behavior and mild depressive symptoms. We intend to enroll 40 individuals in our study. Screened and eligible participants will perform baseline assessments and provide a blood sample. Next, they will be randomized to consume either a blueberry powder (which provides ~ 8 g/day of fiber and ~ 600 mg/day of anthocyanins) or placebo powder (nutritionally matched powder without anthocyanins or fiber) for 3 months (Fig. 1). The study was approved by the Advarra IRB (#Pro00064749). The protocol that is described in this paper is Version 8, dated 2/1/2024. The study was registered at Clinicaltrials.gov (Identifier: NCT05735587). The SPIRIT-Outcomes 2022 Checklist was used to ensure all relevant information regarding the study is included in this paper.

### Study objective and hypothesis

Our objective is to determine feasibility of a freeze-dried blueberry powder intervention and its preliminary efficacy on our proposed outcomes of inflammatory markers, motivation, and physical activity engagement. We hypothesize at least 80% of the participants enrolled will complete the 3-month intervention, and that levels of relevant inflammatory markers will decline, and physical activity will increase in the intervention compared to the control supplement. We further hypothesize that at least 10% of the impact on physical activity is mediated via an increase in self-reported motivation.

### Study population and recruitment plan

Our target population is older adults aged ≥ 65 years with sedentary (self-reporting ≥ 8 h of sitting per/day) behavior and minor depressive symptoms (defined as ≥ 4 and < 16 points on the center for epidemiological studies depression-scale, CES-D [30, 31] or the CESD-revised scale, CESD-R [32]). We plan to recruit individuals with minor depressive symptoms since these individuals typically have higher levels of inflammation [33–35] and often experience lack of motivation to engage in physical activity [15, 16, 36]. Such individuals would theoretically stand to reap the most benefit from a therapeutic strategy that lowers inflammation and improves motivation. In 2023–2024, individuals in the greater Boston area expressing an interest in participating after recruitment out-reach engage in a preliminary telephone screen, and if eligible continue with an in-person screening. We intend to enroll 40 individuals. Recruitment strategies include local newspaper and internet advertisements, physician referrals, registries of research volunteers, and presentations at senior housing sites in the greater Boston area. We also plan to hire a recruitment company *(e.g.,* BuildClinical) that uses data-driven approaches and presents internet advertising to local people whose search history suggests they may be interested and eligible.

### Exclusion criteria

Exclusion criteria have been selected to ensure safety and optimize compliance, while minimizing confounders due to overt disease or conditions that may significantly influence study outcomes. The specific exclusion criteria are listed below:

- Unwilling to follow study protocol
- A median daily step count > 7,500 steps per day (as measured by the ActiGraph), which indicates the older adult is meeting the recommended levels of moderate/vigorous physical activity [9], or per discretion of the PI
- Cognitive impairment (defined as Montreal Cognitive Assessment, [37] MoCA < 22 points)
- Self-reporting a history of inflammatory bowel disease/syndrome, major depression, bipolar, schizophrenia, or other psychotic disorders, or per discretion of the PI
- Self-reporting type 1 or type 2 diabetes
- Allergic to intervention or control products
- Recent use (within the last 3 months) of antibiotics or pro-biotics, or per discretion of the PI
- Current substance use disorder (Drug Abuse Screening Test, [38, 39] DAST-10 > 2 points)
- Current alcohol use disorder (Alcohol Use Disorders Identification Test—Consumption, [40, 41] AUDIT-C ≥ 4 points)
- Unstable anti-depressant use (e.g., change in medication within last 3–6 months), or per discretion of the PI
- Current homicidal or suicidal ideation (assessed via the P4 Suicidality Screener [42])
- Current psychosis (via the Psychosis and Hallucinations Questionnaire, [43] PHQ > 12 points)
- Manic symptoms (assessed by the Mood Disorder Questionnaire, [44] MDQ > 5 points)

### Informed consent and confidentiality

All interested individuals will be asked to provide verbal consent to complete an initial eligibility screen during a phone conversation with study personnel. Potentially eligible participants will then schedule an in-person screening visit. Potential participants may be sent by email or conventional post (per request, and according to their preference) a copy of the informed consent form for them to review at their own pace prior to the in-person screening. Written informed consent will be obtained by study personnel at the beginning of the in-person screening visit.

The following are the planned procedures for effectively protecting against and minimizing loss of participant privacy:

1. Phone screening will be conducted in a private office space.
2. Study visits will be conducted in private rooms.
3. Each participant will be given a unique study identification number and data will not include any of the participant’s protected health information (PHI).
4. All participant-identifying information will be stored and managed on a secured database server. The information will be password protected.
5. Participant confidentiality will be maintained in accordance with Health Insurance Portability and Accountability Act (HIPAA) regulations.
6. Only the PI, study personnel, and laboratory personnel approved by the IRB and authorized to view PHI will have access to the information.
7. PHI will not be used during discussion, presentation or publication of any research data.
8. Files containing PHI data collected for recruitment and screening purposes will be kept in locked, secured filing cabinets accessible only to designated study personnel (research assistants and investigators)

### Proposed intervention

The entire duration of this study is a total of 14 weeks. During the entire 14 week study period, participants are asked to avoid eating certain foods (e.g., fiber- and anthocyanin-rich foods), while also monitoring their usual physical activity with an accelerometer (i.e., an ActiGraph GT9X Link) prior to the intervention period. After two weeks of avoiding pre-specified foods and wearing the activity monitor, participants are randomized to either the intervention or the placebo group. Those randomized to the intervention group are asked to consume approximately 48 g of freeze-dried blueberry powder as a source of ~ 8 g of fiber and 600 mg of anthocyanins. The proposed dose of 48 g of freeze-dried blueberry powder is equivalent to approximately 2 cups of whole blueberries. Individuals randomized to the control group are asked to consume approximately 48 g of a nutritionally matched placebo powder that is devoid of anthocyanins and fiber. Given that the intervention and control products are regularly consumed, we do not anticipate any toxicities.

### Study powders

The blueberry powder and the placebo powder were provided by the United States Highbush Blueberry Council. The blueberry powder consists of harvested whole blueberries that were freeze-dried and ground into a powder form. Thus, the freeze-dried blueberry powder should contain all of the nutrients as a whole blueberry. The placebo powder was nutritionally matched and primarily composed of maltodextrin, fructose, citric acid, as well as other flavoring and coloring agents. However, to our knowledge the placebo powder is devoid of anthocyanins and fiber, which will serve as an appropriate control/comparison, allowing for evaluation of the impact of the putative active ingredients.

### Study blinding

Both powders were provided by the United States Highbush Blueberry Council. The powders are individually packaged in 24 g amounts. The two powders look, taste, and are packaged similarly, however their packaging differs by a symbol. One set of powder was labeled A and the other powder was labeled B. The key that outlines which letter is assigned to the blueberry powder and the letter that is assigned to the placebo powder will be held by the United States Highbush Blueberry Council, who packaged the powders. The key will be kept from study staff and data analyzers until the end of the study and data analysis to maintain blinding of the study team and the participants.

### Compliance monitoring

Participants are instructed to consume 2 packets of their respective powder each day for 12 weeks. It is recommended that participants consume their powder all at once in the morning. To avoid participant fatigue with powder consumption, participants are allowed to consume the powder in multiple modalities (e.g., mixed with water, sprinkled on low-fiber cereal, blended with ice as a “smoothie” etc.), as long as they documented the modality in which the powder was consumed each day. Compliance with our dietary intervention is evaluated by asking participants to fill out daily compliance logs and keep all of their used and unused powder packets. At the study visits, participants return all unused and used powder packets to estimate the number of intended doses that were consumed.

### Randomization scheme

This is a parallel study; therefore, the participants will be randomized to the intervention or the placebo group at the baseline visit. In an attempt to account for the impact of seasonal variation on physical activity levels, we will employ block (blocks of 4) randomization. For every block of 4 participants, 2 will be randomized to consume the placebo powder and the remaining 2 will be randomized to the blueberry powder. SAS will be used to generate a randomized sequence of the two arms with blocks of 4.

### Intended study procedure

Before the 12-week intervention individuals are asked to avoid consumption of high fiber and anthocyanin-rich foods (e.g., blueberries) for 2 weeks. During this time, individuals wear an accelerometer device (i.e., an ActiGraph GT9X Link watch) to objectively measure their usual daily step count (an exclusionary criteria). The median value over approximately 7 days is used as the daily step count metric to determine final eligibility (i.e., participants are eligible if median step count < 7,500 steps/day). After completion of the 2-week washout and 3-day diet records, participants come in for their baseline visit where they are provided with a daily step goal (20% increase of their median daily step count). Participants then complete baseline assessments of vitals, height, weight, social connectedness, motivation, and provide a blood sample for the evaluation of serum inflammatory markers (Table 1). Participants then enter the intervention phase of the study, where they consume their assigned powder, continue to avoid consumption of high fiber and anthocyanin-rich foods, and continue to wear their ActiGraph daily for 12 weeks. After 4 and 8 weeks, participants complete telephone check-ins. At 6 and 12 weeks, participants come in for mid-point and final follow-up visits where the baseline assessments are repeated and compliance is measured. Suicidal ideation, depression severity, and self-reported symptoms are evaluated at every study visit.

**Table 1:** Study visits and assessments

	Baseline	4 Weeks	6 Weeks	8 Weeks	12 Weeks
Suicidal Ideation (via P4SS)	X	X	X	X	X
Depression Severity (via PHQ-9)	X	X	X	X	X
Social Network Questionnaire	X		X		X
ActiGraph Monitoring of Activity	X	X	X	X	X
Relevant Symptoms	X	X	X	X	X
Vitals	X		X		X
Height	X				
Weight	X		X		X
Blood Draw	X		X		X
Serum Inflammatory Markers	X		X		X
Motivation (via MPAM-R)	X	X	X	X	X
3-Day Diet Record	X		X		X
Compliance		X	X	X	X

*MPAM-R* Motives for Physical Activities Measure-Revised (MPAM-R), *P4SS* P4 Suicidality Screener, *PHQ-9* Patient Health Quesionnaire-9

### Expected primary outcome variables

The primary outcome for this study is engagement in physical activity. To our knowledge, no trials have been conducted that evaluate the impact of a dietary intervention on change in step count in our specific population. Therefore, this lack of data serves as the rationale for selecting step count as our primary outcome. The variation of change collected from this study will be used to inform the power and sample size calculations for subsequent studies.

#### Engagement in physical activity

Engagement in physical activity is evaluated by the median number of steps per/day (via ActiGraph) of the previous two weeks before each visit (e.g., 2 weeks before baseline or 2 weeks before follow-up). We also plan to evaluate other aspects of physical activity (e.g., number of sedentary minutes/day) in a similar fashion.

### Expected secondary outcome variables

Other secondary outcomes include:

1. *Feasibility*: Feasibility is evaluated as participant retention rate (i.e., number of participants that complete the intervention/total randomized), however other aspects will be explored.
2. *Inflammatory Biomarkers*: Serum markers relevant to inflammation, (serum brain-derived neurotrophic factor, BDNF; C-reactive protein, CRP; interleukin-6, IL-6) are measured at baseline, mid-point (6 weeks) and final follow-up (12 weeks).
3. *Motivation*: Motivation to engage in physical activity (the proposed mediator between inflammation and sedentary behavior) is assessed by the self-report questionnaire Motives for Physical Activities Measure-Revised that has been used in middle-aged and older adults [45].

### Other variables

 Below are specific protocols outlined for assessments beyond primary and secondary outcomes.

- Medical History/Health Behaviors: Additional measures to characterize the participants include existing or previous medical conditions, current medications, smoking status, use of alcohol, etc.
- Social Network: Subjective perception of social support and connectedness is evaluated by the Multidimensional Scale of Perceived Social Support questionnaire [46]. This assessment consists of 12 items that evaluate an individual’s perceived adequacy of social support from family, friends, and significant others.
- P4 Suicidality Screener is a validated 4-item questionnaire that assesses suicidal ideation [42]. It has been used in several trials as an assessment in adults between the ages of 55–73 years [47–49]. This is a questionnaire that is administered to participants by a trained staff member. Responses that indicate a potential suicidal risk triggers further assessment of imminent risk of the participant. Any participant that presents as a potential suicide risk is referred to a local suicide risk hotline (e.g., The Samaritans of Boston: 617-247-0220). Study staff are trained to call 911 for immediate assistance if a participant indicates he/she has a plan to commit suicide and is perceived as a serious and/or dangerous situation.
- P4 Suicidality Screener is a validated 4-item questionnaire that assesses suicidal ideation [42]. It has been used in several trials as an assessment in adults between the ages of 55–73 years [47–49]. This is a questionnaire that is administered to participants by a trained staff member. Responses that indicate a potential suicidal risk triggers further assessment of imminent risk of the participant. Any participant that presents as a potential suicide risk is referred to a local suicide risk hotline (e.g., The Samaritans of Boston: 617-247-0220). Study staff are trained to call 911 for immediate assistance if a participant indicates he/she has a plan to commit suicide and is perceived as a serious and/or dangerous situation.
- Patient Health Questionnaire-9 (PHQ-9) is a 9-item, self-report questionnaire about feelings or problems that may affect feelings that is used to categorize participants into a depression level category (normal, mild, moderate, moderately severe, or severe). As a safety precaution, the participant’s depression level is monitored via the PHQ-9 which has relatively high sensitivity and specificity [50]. At any time, if an individual scores >9 points (indicating more severe forms of depression), then they are asked to speak with the study psychiatrist to determine whether they should continue on the study. Any reports from the PHQ-9 assessment >15 points (indicating moderately severe forms of depression) qualify as an adverse event (AE).
- Relevant Symptoms: Information on relevant symptoms including gastrointestinal distress, appetite, pain etc., is collected by self-report at each study visit. Any reports of new symptoms that are moderate in intensity qualify as an AE.
- 3-Day Diet Records: To evaluate dietary intake over the course of the study, diet records (consisting of 2 weekdays and 1 weekend day) are collected from participants and reviewed by research staff for accuracy and completeness. Records are entered into a dietary analysis program (e.g., Nutrition Data System for Research) to estimate dietary intake of nutrients.
- Vital﻿s, Height and Weight are measured to help characterize the participants.
  1. Vital signs (e.g., body temperature, pulse, and seated blood pressure) are measured at each in-person visit baseline visit. After 3–5 min of rest, seated blood pressure is measured twice with an automated cuff
  2. Height is measured using a stadiometer.
  3. Weight is measured using a digital Health-o-meter scale.
- Blood (up to 10 mL) is collected by a trained phlebotomist using sterile procedures. Blood is centrifuged at 3.6 x 1000 RPM for 15 minutes at 4 °Centigrade for EDTA plasma or after 30 minutes of clotting at room temperature at 3.6 x 1000 RPM for 10 minutes at 4°Centigrade for serum. Aliquots of serum/plasma are then stored at −80 degree °Centigrade for future analyses of relevant inflammatory markers.

### Plans to promote retention

At the start of an individual’s study participation, he/she will be given a schedule of their study visits if desired. Visits will be scheduled at a time of day that the participant determines is most convenient for them, and will aim to be repeated at the same time for each subsequent visit. Transportation will be provided for each visit as needed, snacks will be available, and stipends will be provided for each study milestone. If necessary, reminder calls will be made to participants on approximately 2 days prior to study visits. Notes that may facilitate compliance, such as “call before 10 am,” etc., will be kept in participant files. We will employ specific strategies to maximize participation and compliance:

- *Positive Framing about Benefits:* Information will be presented in terms of the possible gains rather than the avoidance of losses as this is a more effective motivational approach.
- *Feedback and Recognition of Progress:* Participants will be acknowledged throughout their participation and will be recognized for their contributions to the study. We will remain in close contact with individuals throughout their participation with regular follow-up calls.
- *Incentives and Rewards:* Participants will receive snacks at each visit, cards for achieving milestones, such as birthdays, holidays, etc.; and certificates of completion.

If a participant expresses dissatisfaction with the intervention products and requests a changes in the amount they are expected to take, they will be advised to consume as much of the full dose as they can but consume at least 1 packet of powder (i.e., 24 g/day). If a participant does drop out of the study after receiving the intervention, there are no plans to continue with assessments/visits.

### Collecting and reporting of adverse events

All adverse or serious adverse events (AE, SAE) will be logged using forms either provided by or modeled after the forms that are provided by the NIA Clinical Research Toolbox. The PI, study psychiatrist, and/or trained staff member will evaluate all adverse events as to their severity (i.e., mild, moderate, severe, life threatening) and relation (i.e., unlikely, possibly, probably) to the test article. A serious adverse event is any experience that results in either, death, a life-threatening situation, or in-patient/prolonged hospitalization. All AEs/SAEs will be reviewed by the PI and reported to the IRB as appropriate. All other adverse events/study incidents will be logged on an Adverse Event log and reported to the IRB following the appropriate reporting times as defined by the Advarra IRB.

Any AE’s that 1) are unexpected in nature, severity, or frequency, 2) are possibly, probably, or definitely related, and 3) suggests that the research places participants at a greater risk of harm than previously known or recognized, will be reported to the IRB, NIA PO, SO, and OHRP within 2 weeks of the event. Study staff will reference a Subject Safety Event Reporting Decision Chart provided and updated regularly by Advarra to determine whether an event needs to be reported to the Advarra IRB.

Given that our population has mild levels of depressive symptoms, we have defined specified thresholds of change in some of our assessments (e.g., change in related symptom severity or change in depressive symptom severity) that will qualify as an adverse event. At baseline visit, we collect information on self-reported symptoms. Reports of a symptom at the baseline visit will not be reported as an adverse event since this symptom may have existed prior to the intervention. These measures will serve as our reference measure to determine whether a subsequent assessment will warrant an AE report. For example, if a participant reports presence of fatigue at baseline visit, reporting fatigue at subsequent visits will not qualify as an AE unless the participant exclaims the symptom is more severe than usual. Newly appeared symptoms that are documented as moderate or severe will be documented as AE’s. Other possible adverse findings could be related to assessments such as vitals measurement or depressive symptoms. For safety, we will evaluate the level of depression severity utilizing the validated questionnaire, PHQ-9 [50]. At any time, if the individual scores > 9 points (indicating more severe forms of depression), then they will be asked to consult the study psychiatrist to determine whether they should continue on the study. Any reports from the PHQ-9 assessment ≥ 15 points (indicating moderately severe forms of depression) will qualify as an AE. Similarly, if at any time the participant presents with a seated blood pressure above 180/100 mmHg or a pulse > 100 this will qualify as an AE and the study staff will inform the PI, who will then notify the primary care physician.

### Data safety monitoring

Since this is a single-site, phase 1 pilot study, without high risk, our study will not require an official Data and Safety Monitoring Board (DSMB). However, to ensure and monitor participant safety, a study psychiatrist will oversee all possible adverse events and we will also have a Safety Officer (SO) designated for this study, who resides in the Boston area. The PI, study psychiatrist, and SO will meet at least twice per year to review study progress, data quality, and participants safety. Prior to the intervention period, SO will review the entire IRB-approved study protocol regarding subject safety and analysis, the informed consent documents regarding applicability and readability, and participant recruitment and retention milestones.

### Participant stopping rules

If a participant experiences any adverse event that is deemed “severe” their continuation in the study will be determined by the PI. If necessary, the PI will consult the study psychiatrist and/or SO to gain additional insight on participant continuation. Additionally, if a serious adverse event (SAE) occurs, it will be carefully reviewed by the SO. Any report of a serious adverse event (SAE) that is thought to be directly related to the study products or study procedures, will result in the participant’s discontinuation from the study.

### Study stopping rules

Similar to the participant stopping rules, all serious adverse events (SAE) will be carefully reviewed by the SO to determine if study termination is warranted.

### Data management

 All data collected for analysis is de-identified and assigned a unique study number. Any data collected on paper forms is kept in a locked file cabinet at HRC. Data collected on paper forms is entered and stored on a password-protected secure server at HRC. When possible, data is collected directly via our electronic data capture system (e.g., REDCap).

### Statistical analysis: sample size, statistical power, and analysis

The sample size for this study was chosen to provide a practical basis for developing variance measures of motivation and engagement in physical activity that are needed to design a subsequent larger randomized controlled trial. Thus, due to the preliminary nature of this pilot study, no formal power analysis was performed. However, a power estimation for the daily step count outcome was computed. We estimated the standard deviation of change in steps utilizing the variation of steps in older, sedentary adults without depressive symptoms previously published [51]. Assuming SD of change = 1045, 80% power, and an alpha = 0.05, a sample size of 18/group is required to detect a change of 1000 steps/day. An increase in 1000 steps per day was associated with a 16% reduced risk in all-cause mortality [51]. The estimated sample size of 18/group could be achieved providing we meet our recruitment goal of 40 individuals, and they complete the entire intervention.

All analyses will be conducted with the intent to treat, and explored per protocol. Formal inference and estimation of treatment effects will use a Student’s t-test. To assess change in the primary outcome variable between groups, absolute change (baseline – follow-up) in 2-week median step count will be calculated for each individual. This change in step count will be compared between the experimental (blueberry powder) and the control group (placebo powder) using a Student’s unpaired t-test. Other outcomes will be analyzed in a similar fashion: mean change (baseline – follow-up) in the outcome variables (e.g., inflammatory markers, motivation) in the control group will be compared to the mean change (baseline – follow-up) of the intervention group overtime. We will also consider change between baseline and 6 weeks (midpoint) for the outcomes to explore the rate of change of these variables using a repeated measures analysis. A p-value less than 0.05 will be used to determine statistical significance. We also plan to evaluate the relationship between of change in inflammatory markers with change of physical activity utilizing a linear regression. To determine if change in motivation is on the pathway between change in inflammation and engagement in physical activity, we will use linear regression with and without the addition of change in motivation to the model. If adjusting for change in motivation changes the estimate by > 10% (this threshold is commonly used in the change in estimates approach [52–54], then motivation will be considered a part of the causal pathway. A p-value less than 0.05 will be used to determine statistical significance.

Sub-group analyses are planned for select groups (e.g., those who are compliant with our intervention, those not using anti-depressants, those using anti-depressants, those with more severe depressive symptoms at baseline).

### Berries, Bugs and the Blues, a sub-study

All individuals enrolled in the Berries and Steps study are offered an opportunity to enroll in a sub-study called Berries, Bugs and the Blues that is funded by the USDA (2022–09412), approved by the Advarra IRB (#Pro00068528), and registered at Clinicaltrials.gov (Identifier: NCT05817383). The objective of the sub-study is to evaluate the impact of daily blueberry consumption on the gut microbiota, gut-derived metabolites (i.e., short chain fatty acids), and depressive symptom severity. All individuals provide consent for the use of data/biological samples for this study. Individuals that enroll in the sub-study are asked to provide a stool sample at baseline and at final follow-up (week 12). Participants collect their stool sample in the comfort of their own home approximately 3 days before their baseline visit. The sample is collected in a sterile tube and temporarily placed in the participant’s freezer until transferred for long-term storage at −80 °C. At the end of the study, samples will be sent out to a reputable lab for measurement of gut-derived metabolites (e.g., short chain metabolites) and whole genome sequencing. Additionally, participants are asked about sleep quality (via Pittsburg Sleep Quality Index [55]), anxiety (via Generalized Anxiety Disorder-7 questionnaire [56]), attitude (via Positive and Negative Affect Scale [57]) and severity of depressive symptoms (via CES-D [30, 31] and the CESD-revised [32]) at baseline, week 4, week 8, and final follow-up.

### Plans to communicate findings

Preliminary findings that can be analyses without breaking the blind (e.g., feasibility metrics) may be reported/presented at scientific conferences. Results may be reported to clinicaltrials.gov, as required. Regardless of the findings, we plan to report our analyses in an open access journal following data analysis.

## Discussion

Despite consistent evidence that diet modulates inflammation [17–21], and inflammation is linked to lack of motivation [10–12], to our knowledge no dietary interventions have supplemented anti-inflammatory nutrients to target motivation and thereby increase physical activity in older adults. Given that sedentary behavior has grave consequential health outcomes in older adults, a dietary intervention that is designed to improve engagement in physical activity by targeting inflammatory pathways of motivation holds great promise.

Fiber and anthocyanins are known for their anti-inflammatory capacity, and may be a viable supplemental strategy to reduce inflammation and improve motivation to engage in physical activity in older adults. Since fruits and vegetables are a rich source of these nutrients, fiber and anthocyanins can be easily obtained at grocery stores and are widely accessible to a large proportion of older adults. Older adults typically consume small quantities of both anthocyanins [58] and fiber, [27] which may not be sufficient to harness their anti-inflammatory benefits. A plethora of interventions supplementing berries/berry products (a concentrated source of fiber and/or anthocyanins) have been shown to reduce markers of inflammation in various populations (age range: 20–70 y, including healthy individuals and those with diabetes, obesity, osteoarthritis etc.) [59–68], Older adults with depressive symptoms, who typically have high levels of inflammatory cytokines [69], may be particularly responsive to an anti-inflammatory dietary intervention.

However, it is well described that changing an individual’s dietary pattern can be difficult, particularly for older adults who are typically accustomed to an established dietary routine. Therefore, many studies opt to incorporate a single daily food or nutrient supplement to avoid the burden of changing an entire dietary pattern. The current study was designed to provide a daily source of fiber and anthocyanins via a powder. This strategy allows the study to be blinded since both experimental and control powders will be packaged in the exact same way, as well as look and taste similar. Although the blueberry powder is made from whole blueberries by merely removing the water content and in theory should have the same nutrients as a whole blueberry, some nutrients may have degraded during this process. Therefore this study can only provide indirect evidence of the impact of whole blueberries on the measured outcomes. Furthermore, we acknowledge that the consumption of powder every day for 12 weeks may result in fatigue and lead to low compliance or discontinuation of the study. Therefore, we plan to provide participants with suggestions/recipes on how to consume the powder (for example, mixed in 12 fl oz of water or mixed in 12 fl oz of almond milk). At the beginning of the study, participants are given cooking appliances (e.g., mixing apparatus) or ingredients (e.g., almond milk) that can be used to make suggested recipes. This strategy of allowing for other modalities of intake may also improve compliance with the amount of powder participants are asked to consume. Most studies ask participants to consume approximately 24 g of powder (equivalent to 1 cup of blueberries). Here, we ask participants to consume 48 g, which provides our theoretically effective dose of anthocyanins and fiber. Even if participants are unable to consume the entire dose, they will be encouraged to consume as much as they can, which will allow us to perform an intent to treat analysis. Research staff completes frequent check-in calls with participants i to find a suitable method to facilitate compliance with the protocol as intended.

Although electronic data capture devices collect objective measures, using them in research also comes with its own challenges. In order to collect physical activity data from participants the current study utilizes an accelerometer (i.e., the ActiGraph GT9X Link) that participants are asked to wear for the entire duration of the study. A limitation of this specific model is that is does not have the capacity to transmit the data to a cloud-based server, thus prohibiting real-time review of data. Instead, the research team is required to retrieve the physical device to then download the data, which is time consuming. Future studies may want to consider using devices that are able to wirelessly sync data to a cloud based server.

Since studies have not yet evaluated changes in step counts in response to an anti-inflammatory dietary intervention in our specific population (older, sedentary adults with mild depressive symptoms), the variations of change that are gathered from this study will be used to estimate power for large scale efficacy trials. As such, no formal power analysis was done to determine the sample size needed to show significant differences in our outcomes. However, study feasibility is one of the secondary outcomes of this study, which will allow us to estimate the number of individuals we need to screen and enroll in order to achieve a desired sample size. Together, our outcomes, both the feasibility findings and the data on variation of change, will be informative for the design of a larger, more robust subsequent trial designed to establish definitive efficacy of a fiber and anthocyanin intervention to increase physical activity in sedentary older adults.

## Data Availability

No datasets were generated or analysed during the current study.

## Abbreviations

CES-D: Center for epidemiological studies depression-scale
CESD-R: CES-D revised scale
MoCA: Montreal cognitive assessment
DAST-10: Drug abuse screening test
AUDIT-C: Alcohol use disorder identification test—consumption
PHQ: Psychosis and hallucinations questionnaire
MDQ: Mood disorder questionnaire
PHI: Protected health information
HIPAA: Health Insurance Portability and Accountability Act
MPAM-R: Motives for Physical Activities Measure-Revised
P4SS: P4 Suicidality Screener
PHQ-9: Patient Health Quesionnaire-9
BDNF: Brain-derived neurotrophic factor
CRP: C-reactive protein
IL-6: Interleukin-6
AE: Adverse event
SAE: Serious adverse event
PI: Principal investigator
SO: Safety officer
PO: Program officer
NIA: National Institute of Aging
DSMB: Data and Safety Monitoring Board
USDA: United States Department of Agriculture
